# Point-of-Care Ultrasound Evaluation of Diaphragm as a Predictor of Extubation Success in Mechanically Ventilated, Malnourished, End-Stage Liver Disease Pretransplant Candidates—Observational Cohort Study

**DOI:** 10.1177/29768675251337833

**Published:** 2025-05-02

**Authors:** Adam Muhammad Reid, Sarathi Bhattacharyya, Zian Zhuang, Nida Qadir, Amy Schnabel, Alan Chiem, Semi Yoon, George Lim, Igor Barjaktarevic

**Affiliations:** Department of Internal Medicine, 6566 Jefferson Einstein Philadelphia Hospital, Philadelphia, Pennsylvania, USA; Department of Medicine, Long Beach Memorial Medical Center, Long Beach, California, USA; Department of Biostatistics, Fielding School of Public Health at UCLA, Los Angeles, CA, USA; Division of Pulmonary, Critical Care, and Sleep Medicine, 12222 David Geffen School of Medicine at UCLA, Los Angeles, CA, USA; Clinical Nutrition, Liver Transplant service, Ronald Reagan Medical Center at UCLA, Los Angeles, CA, USA; Department of Emergency Medicine, 12222 David Geffen School of Medicine at UCLA, Los Angeles, CA, USA; Division of Pulmonary, Critical Care, and Sleep Medicine, 12222 David Geffen School of Medicine at UCLA, Los Angeles, CA, USA; Department of Anesthesiology & Perioperative Medicine, 12222 David Geffen School of Medicine at UCLA, Los Angeles, CA, USA; Division of Pulmonary, Critical Care, and Sleep Medicine, 12222 David Geffen School of Medicine at UCLA, Los Angeles, CA, USA

**Keywords:** diaphragm thickening fraction, ultrasound, POCUS, weaning from mechanical ventilation, extubation failure, malnourished, debilitated, cirrhosis, end-stage liver disease

## Abstract

**Background:**

Ultrasound evaluation of diaphragmatic anatomy and function has recently gained traction as a simple and useful tool to assess the extubation readiness in mechanically ventilated patients, nevertheless, how applicable this approach is in the population of chronically debilitated patients on mechanical ventilation (MV) remains unclear.

**Objective:**

To evaluate ultrasonographic assessment of diaphragmatic thickening fraction (ΔTDI%) as a predictor of extubation success in the population of end-stage liver disease (ESLD) malnourished patients on MV.

**Design:**

Prospective, single-center, observational cohort study

**Methods:**

We used point-of-care ultrasound to evaluate ΔTDI% and diaphragm thickness during expiration (*T*_exp_) and inspiration (*T*_insp_) as predictors of extubation success in ESLD patients undergoing weaning from mechanical ventilation. The primary end-point was extubation tolerance (ET) assessed at 48 h.

**Results:**

Of 70 enrolled patients, 82.4% (*N* = 56) tolerated extubation. While there was no difference in ΔTDI% between those who failed extubation (EF) compared to ET at 48 h (21.2% vs 20.1%, *P* = .64), diaphragms were thicker at expiration in ET patients (*T*_exp_ 29.5 ± 8.1 vs 24.8 ± 5.2 mm, *P* = .047). Commonly used clinical weaning parameters, including rapid-shallow breathing index (RSBI) and negative inspiratory force (NIF) correlated better with diaphragm thickening fraction ΔTDI% than diaphragm thickness indices but were inferior predictors of extubation success compared to *T*_exp._.

**Conclusion:**

Point-of-care ultrasonographic assessment of the diaphragm offers insight into the function of respiratory muscles and the limited ability to predict extubation success. Further research is necessary to better understand its potential use in MV liberation in patients with ESLD and malnutrition.

## Plain Language Summary Title

**Ultrasound assessment of diaphragm to predict extubation success in mechanically ventilated malnourished end-stage liver disease patients:**

**Plain Language Summary:**

Predicting the ability to tolerate discontinuation of mechanical ventilation in patients with chronic medical conditions and acute respiratory failure is difficult as a large portion of these patients end up needing to be placed back on the respirators. Bedside ultrasonography represents a novel and promising tool helping physicians to better recognize which patients may have more difficulty to tolerate extubations, nevertheless little is known about how useful this approach may be in the population of debilitated and malnourished patients with end-stage liver failure. In order to answer this question, this observational study fails to identify the breathing-induced thickening of diaphragm muscle as a predictor of extubation tolerance, nevertheless identifies the decreased expiratory diameter of the diaphragm to be associated with extubation failure, confirming the relevance of ultrasound in the process of weaning from the mechanical ventilation.

## Introduction

Despite the increasing use of ventilator liberation protocols,^1^ extubation failure, defined as the inability to sustain spontaneous breathing after removal of the endotracheal tube, is a relatively frequent complication in critically ill patients on mechanical ventilation (MV). Liberation from MV is a crucial part of caring for critically ill patients. Delaying extubation results in unnecessary discomfort, increased risk of complications, higher mortality, and increased costs of care.^2^ Despite the known pitfalls of prolonged invasive MV, up to 50% of the duration of MV is spent on the weaning process.^3^ Extubation and transition to negative pressure ventilation induces cardiac, pulmonary, neurologic and psychologic stress on a patient and even with the careful clinical assessment, >10% of extubations fail,^4^ and as many as 20% of extubated patients require reintubation within 72 h.^5^ Extubation failure is associated with prolonged MV, resulting in complications such as barotrauma, ventilator-associated pneumonia, ventilator-induced diaphragmatic atrophy, extended ICU stays and higher hospital mortality.^5,6^ Evaluation of a patient's readiness to be weaned from invasive MV includes assessment of numerous clinical and ventilator-derived parameters. Spontaneous breathing trial (SBT) is one of the major diagnostic tests to determine if patients can be successfully extubated and serves as a tolerance test, allowing those who do not experience neurologic, respiratory, or hemodynamic instability to be candidates for extubation.^7,8^ Rapid-shallow breathing index, respiratory rate, tidal volumes, negative inspiratory force (NIF), secretions amount and gag reflex, muscle strength assessment, or ability to follow commands represent some of the routinely used parameters allowing for a better assessment of the ability to tolerate extubation.^7^

Besides traditional methods assessing patients’ clinical characteristics and their interaction with the ventilator, diaphragmatic ultrasound has emerged as a rapid, noninvasive, inexpensive, and easily reproducible imaging modality capable of assessing respiratory muscle anatomy and function, and has been increasingly studied as a promising tool able to predict extubation readiness.^9–11^ It simultaneously evaluates multiple aspects of respiratory muscle function including the contractility, excursion of the diaphragm as well as the thickness of the diaphragm muscle itself.^12^ The diaphragm thickening fraction (ΔTDI%) has been shown to be a highly useful tool in the weaning from MV with high accuracy with an estimated 77% sensitivity at 80% specificity to predict the extubation success.^13^ While available data find its use in the general ICU population promising, little is known about the usefulness of ultrasonographic evaluation of diaphragm in malnourished and chronically debilitated patients.

The optimization of extubation success predictability may be of particular relevance in patients with cirrhosis and end-stage liver disease (ESLD), as they are often characterized by malnourished state, severe deconditioning, hydrothoraces, and ascites—clinical features that may impact the interpretability of traditional weaning methods.^14–16^ The objective of this study was to evaluate whether direct visualization of diaphragmatic anatomy and function via ultrasound may have advantages in predicting extubation failure compared to more commonly used clinical methods. We hypothesized that diaphragm ultrasound can be used to predict extubation failure in mechanically ventilated, malnourished ESLD pretransplant patients.

## Methods

We conducted a single center, prospective, observational trial to assess the feasibility and clinical utility of diaphragmatic ultrasound to predict extubation success in mechanically ventilated, patients with end-stage liver failure and malnutrition awaiting orthotopic liver transplantation (OLT). The study participants were enrolled at a large liver transplant academic center from 2018 to 2020 and the study was approved by Institutional Review Board (IRB#18-000172). The study size was a convenience cohort of eligible patients enrolled during the predesignated study period. Study methodology was prepared so as to be able to clearly report our data according to the Strengthening the Reporting of Observational Studies in Epidemiology (STROBE) guidelines for cohort studies.^17^ Eligible patients were admitted to the liver transplant intensive care unit, had documented ESLD, and were intubated and mechanically ventilated. The inclusion criteria required moderate to severe malnourished status based on the formal assessment by a Registered Dietitian (A.S.) using the AND/ASPEN Consensus Criteria,^18^ as well as the patient to be undergoing spontaneous breathing trial (SBT) and considered eligible for extubation within 12 h based on the decision made by primary physician/treatment team, able to communicate and follow commands and tolerate pressure support (PS) mode with ventilator settings of PS ≤ 10 cmH_2_O and PEEP ≤ 5 cmH_2_O. Study candidates were excluded if they had major abdominal or chest surgery, excluding chest tube placement, within 3 months of enrollment. Patients with known neuromuscular disorders, diaphragmatic paralysis, hemodynamic or respiratory instability (HR > 130 beats/min, SBP > 180 mmHg or <90 mmHg, RR > 35/min), those anticipated to require elective reintubation within 48 h and those unable to follow commands or tolerate spontaneous awakening trials were also considered ineligible to participate, Supplemental Table 1.

### Data Collection

Baseline characteristics obtained prior to extubation included: age, sex, weight and BMI, nutritional status, APACHE II score, SAPS II score, model for end-stage liver disease (MELD) score, documented history of failure to tolerate extubation during current or previous hospitalizations, prealbumin and presence of ascites or hydrothorax. The presence of hydrothorax and ascites was confirmed by bedside ultrasonography. Hydrothorax was considered large if the size of ipsilateral effusions exceeded 50% of pleural space measured at the midclavicular line using most recent anteroposterior radiography in sitting or standing position^19^ and ascites was graded as large if causing marked abdominal distension, or Grade 3 as per published guidelines.^20^ Clinical, physiological, and ventilator parameters collected at the time of ultrasonographic assessment included: muscle strength, evaluated by a 0–5 scoring system based on the Medical Research Council Manual Muscle Testing scale,^21^ thumbs-up test, head lift test (HLT, ability to elevate the head 30° off the pillow), leg raise tests (LRT, leg elevation 30° off the bed), as well as respiratory rate (RR), expired tidal volumes (*V*_te_), partial pressure of arterial oxygen (P_a_O_2_), fraction of inspired oxygen (FiO_2_), pressure support level, positive end-expiratory pressure (PEEP), suctioning frequency, time spent on pressure support in 24 h, adequacy of inspiratory maneuver, presence of cuff leak, vital capacity, negative inspiratory force (NIF), duration of intubation and rapid-shallow breathing index (RSBI), using cut-off of the ratio of RR/*V*_te_ ≤ 105 as a negative predictor of extubation success.^22^

### Ultrasonographic Assessment of Diaphragm Anatomy and Function

**Figure 1.**
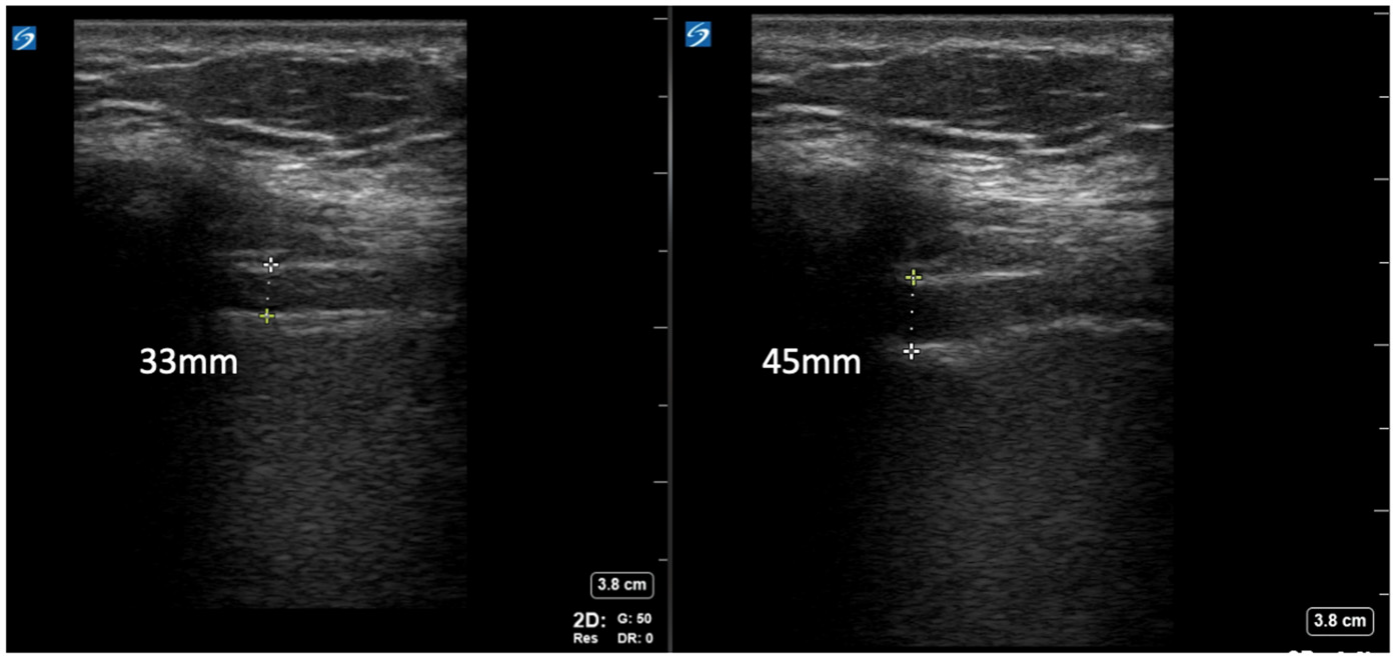
Diaphragm assessment using linear ultrasound probe positioned on the anterior axillary line obtaining B-mode images visualizing diaphragm in: (a) normal expiration and (b) full inspiration. As an example of the measurement of diaphragm parameters – *T*_exp_ = 33 mm, *T*_insp_ = 45 mm, Δ*T* = *T*_insp_  −  *T*_exp_ (45 mm − 33 mm = 12 mm) and ΔTDI% = (*T*_insp_  −  *T*_exp_)*100/*T*_exp_, ((45 − 33)*100/33 = 36.4%). Note that, while M-mode assessment offers easier interpretability and measurements in acquired images, the physical movement of the diaphragm during full inspiration most often would preclude the interpretation of thickness difference in the same spot on the diaphragm, which itself is not of the same thickness in all areas when contracted; using B-mode allows for capturing thickness changes at the same spot, likely improving the accuracy of capturing the thickness changes.

With enrolled participants in a semi-recumbent position with the head of the bed elevated 30–45°, a limited physical exam was performed, ventilatory parameters were collected, and an ultrasonographic assessment was obtained within 12 h prior to extubation. For diaphragm evaluation, a point-of-care ultrasound (POCUS) linear probe (X-Porte, Sonosite, WA, USA) was placed at the right anterior axillary line between the eighth and 10th intercostal spaces with the probe marker pointed cephalad identifying the diaphragm at the zone of apposition. Images were obtained in 2D and M-modes. Diaphragm thickness was measured in millimeters at the end of full inspiration (*T*_insp_) and at the end of normal expiration (*T*_exp_), Figure 1. The diaphragm thickening fraction (ΔTDI%), which represents the percent of the increase in diaphragm thickness during inspiration was calculated using the formula ((*T*_insp_ − *T*_exp_)/*T*_exp_)*100% as previously described,^23^ and the absolute thickening (Δ*T*) was calculated as *T*_insp_ − *T*_exp_. Based on prior studies, we defined subjects with ΔTDI% < 30% as reduced and ΔTDI% > 30% as normal.^11^

### Study End-Points and Outcome Measures

Primary end point was extubation tolerance (ET) or extubation failure (EF) 48 h post-extubation.^2,7,24^ EF was defined as reintubation for respiratory reasons within 48 h post-extubation, which included direct parenchymal disease (eg aspiration, pneumonia, or pulmonary edema) and indirect etiologies of respiratory failure (eg poor airway protection, systemic processes). Patients who were re-intubated electively or for nonrespiratory etiologies (eg gastrointestinal bleeding, intracranial primary events, or electively) were excluded from the final analysis. For the exploratory analysis evaluating ultrasound of the diaphragm as a predictor for extubation success beyond the first 48 h, we assessed patients’ respiratory status at day 7 post-extubation excluding those who did not survive or who had been intubated for elective or nonrespiratory etiologies. The POCUS assessment of ΔTDI% was compared to traditional extubation parameters obtained at the same time.

### Statistical Analysis

Clinical characteristics at baseline and at the time of extubation were compared between the ET and EF groups using the Wilcoxon rank-sum test for continuous variables and the Chi-square/Fisher's test for categorical variables. Sensitivity analysis was also conducted with patients grouped according to their ventilatory status 7 days post-extubation. Spearman correlation was used to investigate the relationship between POCUS and traditional weaning parameters. A univariate receiver operating characteristic (ROC) analysis was used to evaluate the validity of ΔTDI% as a predictor of extubation. Multivariate regression analyses were performed to assess the association between ultrasound parameters and extubation success. Logistic regression models were used for extubation success at 48 h and 7 days, with odds ratios (OR) and 95% confidence intervals (CI) reported. Linear regression was used to evaluate the association between *T*_exp_ and ejection fraction (EF), with regression coefficients (β) and 95% CI reported. All models were adjusted for age, sex, weight, and MELD score, with additional adjustments for malnutrition, prealbumin, ascites severity, and intubation duration as appropriate. Covariates were selected based on clinically reasonable considerations.

## Results

### Study Cohort

Seventy nonconsecutive patients admitted to the intensive care unit, who satisfied eligibility criteria and consented for study participation were enrolled in the study. Study participants were 57.2 ± 12.3 years old with APACHE II scores of 15 ± 4.1, SAPS II 44.5 ± 6.6, and MELD 33 ± 8.9 upon admission. Severe malnutrition was present in 53% of cases and 13% of these patients had a history of extubation failure, Supplemental Table 2.

### Extubation Success

**Figure 2.**
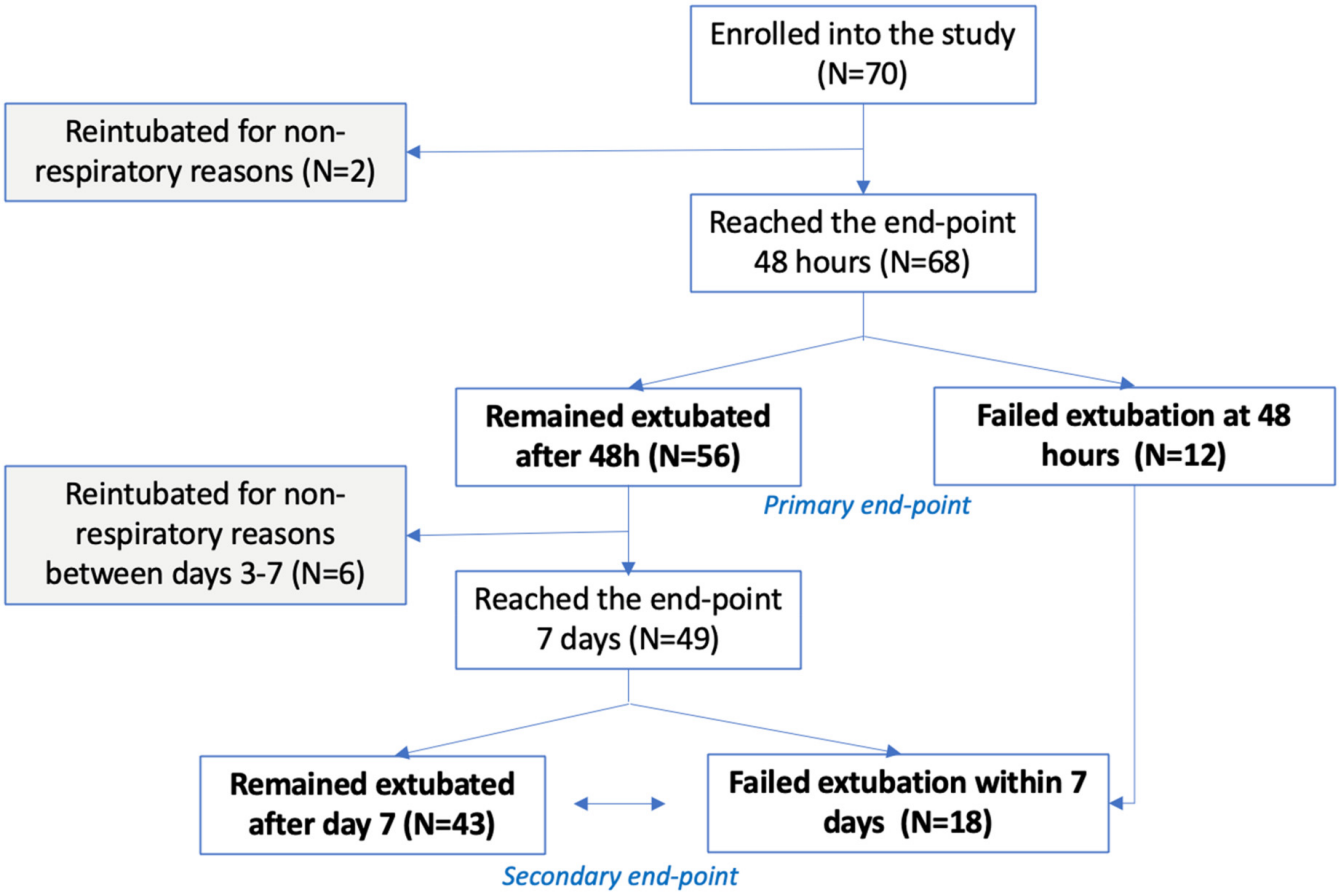
Study consort diagram.

Of patients enrolled in the study, two patients who were re-intubated were excluded from the analysis as they required re-intubation for nonrespiratory reasons. Of the 68 patients who remained in the primary analysis, 12 failed extubation within 48 h, resulting in the extubation success rate of 82.4%. By day 7 post-extubation, an additional 7 patients were excluded from the analysis for reintubation for nonrespiratory reasons. Of the remaining 49 patients, an additional 6 were intubated for respiratory reasons seven days from the extubation, contributing to a total cumulative extubation success rate at seven days of 70.5%. Figure 2.

### Characteristics of Patients Who Failed Extubation

Compared to the ET group, those who failed extubation were younger (age 51.8 ± 12.7 vs 58.1 ± 12.1, *P* = .06), more likely to be male (75.0% vs 44.6%, *P* = .06), and heavier (73.6 ± 14.8 vs 66.8 ± 15 kg, *P* = .09), although these differences did not reach statistical significance. EF patients had large ascites (90.9% vs 39.6%, *P* = .002) and higher white cell blood counts (18.6 ± 13.1 vs 11.3 ± 7.5 cells/µL, *P* = .023), had a numeric trend toward the presence of large ipsilateral hydrothorax and had similar duration of intubation, APACHE II, SAPS II, and MELD score as those who tolerated extubation (ET), Table 1. The presence of large ascites was associated with decreased odds of ET after adjustments for age, sex, BMI, MELD score, degree of malnutrition, and duration of intubation (OR = 0.06, CI95: 0.01–0.59). Similar to the first 48 h post-extubation, in a sensitivity analysis, patients who failed to remain extubated at day 7 (EF_7_) were more likely to have large ascites (86.7% vs 32.4%, *P* < .01) and higher leukocyte counts (17.3 ± 11.6 vs 10.1 ± 7.0 cells/µL, *P* = .003), Supplemental Table 3.

**Table 1.** Demographic characteristics of the cohort and clinical condition prior to the extubation.

Variable		Failed extubation (*N* = 12)		Tolerated extubation (*N* = 56)		*P* Value
		Mean	SD	Mean	SD	
Patient Characteristics	Age (years)	51.8	12.7	58.1	12.1	.06
	Sex, male, *n* (%)	9 (75%)		25 (44.6%)		.056
	Weight (Kg)	73.6	14.8	66.8	15	.09
	BMI	25.3	4.1	24.7	4.7	.44
	Severe Malnutrition, *n* (%)	6 (50%)		28 (53.9%)		.81
	APACHEII Score	15.4	4.8	14.9	4.1	.89
	SAPS II Score	44	8.5	44.8	6.3	.84
	MELD Score	33	8.3	32.9	9.1	.72
	Prealbumin	26.7	7.2	19.9	10.7	.17
	Primary reason for intubation, *n* (%)					.98
	Altered mental status	3 (25%)		14 (25%)		
	Gastrointestinal bleed	4 (33.3%)		16 (28.6%)		
	Respiratory failure	3 (25%)		16 (28.6%)		
	Other	2 (16.7%)		10 (17.8%)		
	Duration of Intubation (Days)	7.2	4.6	8.2	5.9	.62
	History of extubation failure, *n* (%)	3 (25%)		6 (10.9%)		.195
	Large Ascites, *n* (%)	10 (90.9%)		19 (39.6%)		.002
	Large Hydrothorax, *n* (%)	2 (20%)		3 (6.3%)		.084
	Hemodialysis dependence, *n* (%)	11 (91.7%)		46 (82.1%)		.42
	Sedatives administered within 24 h, *n* (%)	5 (71.4%)		16 (55.2%)		.43
	Blood transfusion within 24 h, *n* (%)	4 (33.3%)		10 (17.9%)		.23
	Vasopressors use, *n* (%)	6 (50%)		24 (42.9%)		.65
	White blood cell count	18.6	13.1	11.3	7.5	.023
Pre-extubation condition	Glasgow coma score	5.5	0.9	5.6	1.1	.31
	Heart rate	92.9	19.5	81.3	15.7	.028
	Oxygen saturation (%)	97.4	2.2	97.2	6.9	.41
	Respiratory rate	18.9	7.7	18.3	5.8	.91
	Partial arterial oxygen pressure (mmHg)	102.2	27.1	100.7	32.3	.93
	Fraction of inspired oxyge*n* (%)	32.1	4	31.1	4.6	.7
	Pressure support level	6.7	2.3	6.9	0.5	.99
	Positive end expiratory pressure (cmH2O)	5	0	5	0.3	1

### Clinical Predictors of Extubation Success

As presented in Table 2, RSBI was not a significant predictor of extubation success at 48 h post-extubation (RSBI in EF 63.8 ± 32.6 vs ET 52.2 ± 27.0, *P* = .31). The ability to perform a head lift test was associated with the ability to tolerate extubation (EF 75.0% vs ET 96.4, *P* = .01). The ability to perform a leg raise test was more common in patients with ET (EF 50.0% vs ET 73.6%, *P* = .11), although this difference did not reach statistical significance. Evaluating the same parameters at 7 days post-extubation, positive LRT was associated with remaining extubated (EF 41.2% vs ET 78.1%, *P* < .01). In a multivariate analysis adjusted for age, sex, weight, and MELD score, positive HLT was associated with successful extubation (OR 1.5–195.9, *P* = .02) at 48 h, while positive LRT predicted success at 7 days post-extubation (OR 6.6, CI:1.6–27.8, *P* = .01).

**Table 2.** Predictors of the extubation success

Variable		Time point: 48 h post extubation					Time point: 7 days post extubation				
		Failed extubation (*N* = 12)		Tolerated extubation (*N* = 56)		*P* Value	Failed extubation (*N* = 18)		Tolerated extubation (*N* = 43)		*P* Value
		Mean	SD	Mean	SD		Mean	SD	Mean	SD	
Traditional extubation assessment	Suctioning frequency (Q hours)	3.5	1.4	4.1	1.5	.4	3.6	1.6	4	1.5	.23
	Time on pressure support in 24 h (hours)	7	4.3	9.7	2.7	.13	8.3	3.5	9.8	3	.78
	Tidal volume (ml)	452	158.5	432.8	142.7	.6	423.6	150.7	430.8	146.3	.88
	Adequate inspiratory maneuver, *n* (%)	10 (90.9%)		49 (90.7%)		.98	15 (88.2%)		38 (90.5%)		.79
	Muscle strength (0–5 score)	3.1	1	3.4	1	.21	3.1	1	3.5	1	.06
	Positive head lift test, *n* (%)	9 (75%)		54 (96.4%)		.01	15 (83.3%)		41 (95.4%		.12
	Positive leg raise test (30 degrees), *n* (%)	6 (50%)		39 (73.6%)		.11	7 (41.2%)		32 (78.1%)		.006
	Rapid shallow breathing index	63.8	32.6	52.2	27	.31	68.4	31.4	51	25.5	.06
	Positive cuff leak, *n* (%)	9 (100%)		48 (96%)		.54	14 (93.3%)		37 (97.4%)		.49
	Vital capacity (ml)	986.6	733.4	1669.0	6018.0	.87	941.6	632.2	1911.9	6905.3	.87
	Negative inspiratory force (cmH2O)	27.3	10.9	29.7	10.6	.39	28.2	9.5	29.2	10.1	.67
	Maximal expiratory pressure (cmH2O)	38.3	16.1	26.1	13.6	.24	36.7	18.9	29	14.7	.43
POCUS testing	Maximal inspiratory diaphragm thickness (mm)	31.3	6.7	38	12	.06	34.8	8.1	38	13.1	.67
	Minimal expiratory diaphragm thickness (mm)	24.8	5.2	29.5	8.1	.047	27.8	6.2	29	8.8	.87
	Absolute thickening (mm)	6.5	3.7	8.5	5.4	.3	7.1	3.6	8.9	5.9	.48
	Diaphragm thickness fraction (%)	20.1	10.3	21.2	8.6	.64	20.1	10.3	21.2	8.6	.33
	Diaphragm thickness fraction > 30%, *n* (%)	3 (25%)		10 (17.9%)		.56	3 (16.7%)		10 (23.3%)		.57

### Ultrasonographic Parameters as Predictors of Extubation Success

The diaphragm was optimally visualized in 98.5% of cases, and the most frequent approach was scanning the right anterior axillary line between the 8th and 10th intercostal spaces. The mean diaphragm thickness during inspiration (*T*_insp_) was 36.8 ± 11.5 mm and during expiration (*T*_exp_) was 28.7 ± 7.8 mm. Mean diaphragm thickening fraction (ΔTDI%) during inspiration was 21.0 ± 8.8%, and only 13 (19.1%) patients had ΔTDI% > 30%.

As presented in Table 2, although there was no difference in ΔTDI% between those who failed extubation compared to patients who remained extubated at 48 h post-extubation, diaphragm thickness was different between the groups. Compared to the EF group, ET participants had thicker diaphragm at expiration (29.5 ± 8.1 vs 24.8 ± 5.2 mm, *P* = .047) and had a numeric trend toward thicker diaphragm at full inspiration (38.1 ± 12.0 vs 31.3 ± 6.7 mm, *P* = .06), Supplemental Figure 1. In a multivariate model adjusted for age, sex, MELD, degree of malnutrition, prealbumin, severity of ascites, and duration of intubation, *T*_exp_ was the only POCUS parameter predictive of EF (Coefficient 0.022, CI: 0.002–0.043). Using >30% ΔTDI% as a cutoff was not predictive of extubation (25.0% vs 17.9% of patients had >30% ΔTDI% in EF and ET groups, respectively).

**Figure 3.**
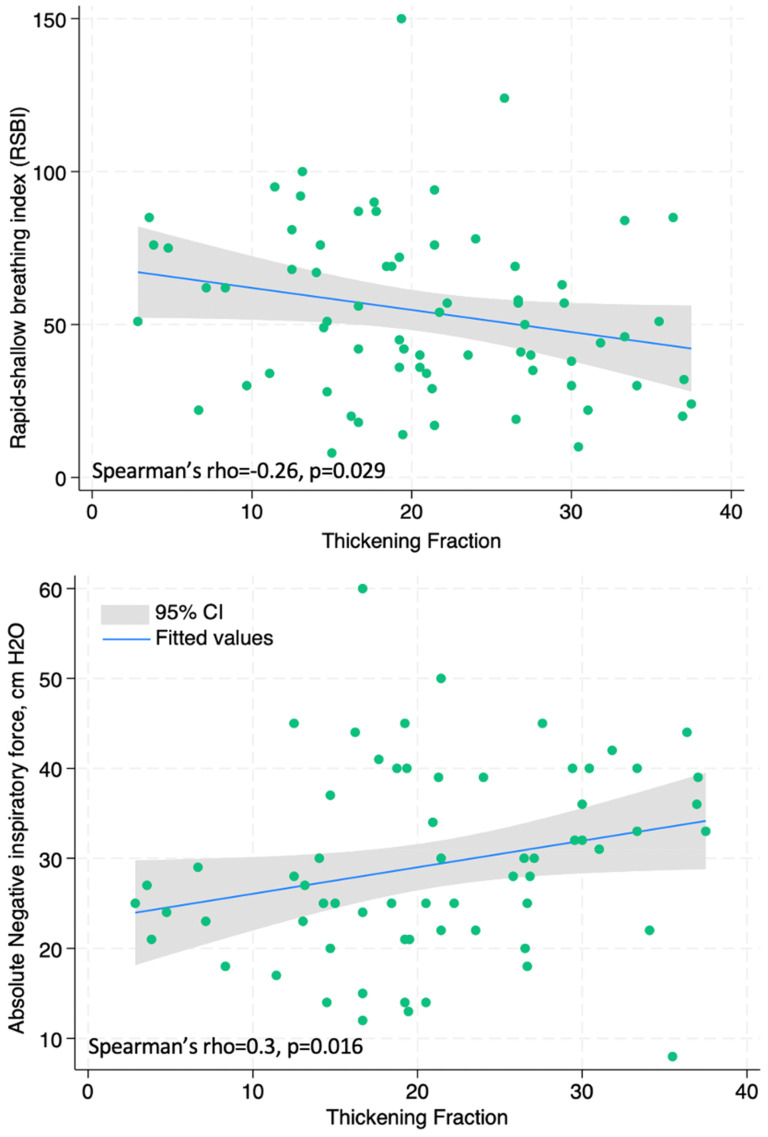
Correlation between ΔTDI% and: (a) RSBI (Spearman's rho = −0.26, *P* = .029) and b) NIF (Spearman's rho = 0.3, *P* = .016).

The relationship of ultrasonographic measures to traditional weaning parameters was also assessed. Both ΔT (Spearman's rho = − 0.29, *P* = .015) and ΔTDI% (Spearman's rho = −0.26, *P* = .029) had negative correlations with RSBI, and ΔTDI% positively correlated with NIF (Spearman's rho = 0.3, *P* = .016), Figure 3.

### Univariate Receiver Operating Characteristic (ROC) Analysis

**Figure 4.**
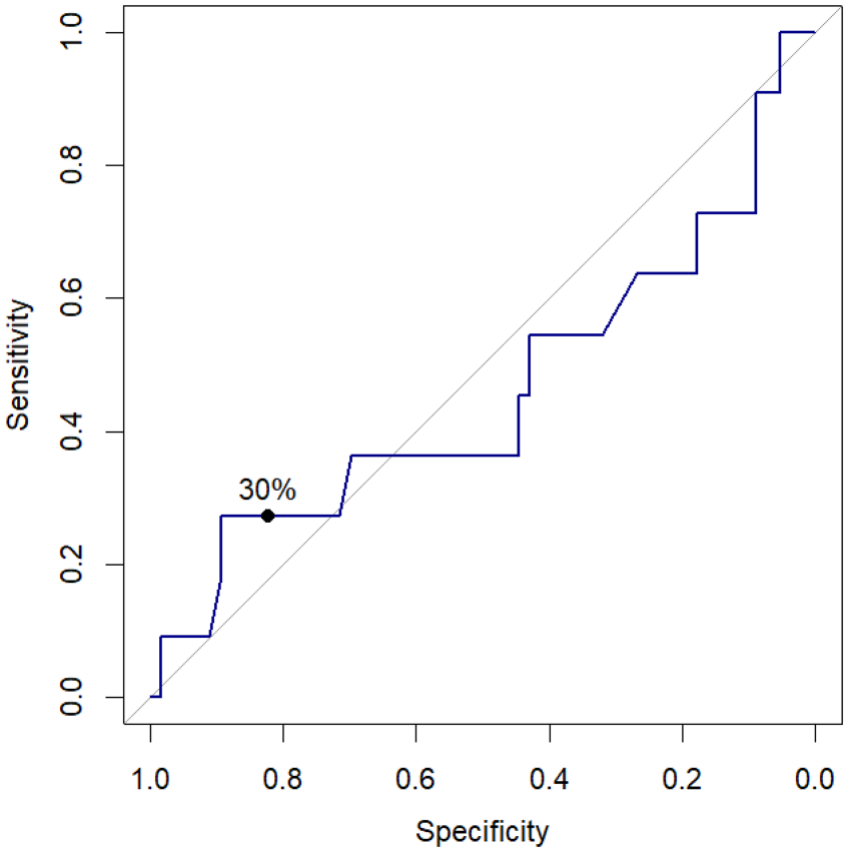
Univariate receiver operating characteristic (ROC) analysis was used to evaluate the validity of ΔTDI% as a predictor of extubation.

As shown in the ROC plot, Figure 4, each point along the curve represents a specific cutoff level along with its associated sensitivity and specificity. We found that the curve is close to the diagonal line, suggesting that the predictor's performance is close to random guessing, as further evidenced by an Area Under the Curve (AUC) value of 0.46. The plot also demonstrates that a cutoff of 30% maximizes the sum of sensitivities and specificities, corroborating previous research utilizing 25%–30% as a threshold to predict extubation success.

### Ultrasonographic Evaluation of the Diaphragm in the Assessment of Nutritional Status and Physical Strength at the Time of Extubation

Compared to those with moderate malnutrition, patients with severe malnutrition had thinner diaphragms (*T*_insp_ 34.1 ± 11.2 vs 40.3 ± 11.8 mm, *P* = .023 and *T*_exp_ 26.9 ± 6.6 vs 31.2 ± 9.4 mm, *P* = .066) and lower ΔT (9.1 ± 4.7 vs 7.3 ± 5.6, *P* = .02). Nevertheless, there was no difference in ΔTDI% between the severe and moderately malnourished patients. Thickening fraction correlated with clinically evaluated muscle strength (Spearman's rho = 0.25, *P* = .045), but none of the ultrasonographic parameters correlated with prealbumin values (*T*_exp_ Spearman's rho = 0.23, *P* = .15).

## Discussion

Evaluating the utility of ultrasonographic diaphragm assessment as a predictor of extubation tolerance in a select population of mechanically ventilated, malnourished patients with end-stage liver disease, we could not identify the diaphragm thickening fraction (ΔTDI%) to be associated with extubation success, nevertheless decreased expiratory thickness of diaphragm was associated with extubation failure. Overall, our results confirm that ultrasound may play a role in the process of weaning from mechanical ventilation, but at the same time stress the importance of the recognition of nuances of the patient population that is being assessed.

Although diaphragm thickening fraction (ΔTDI%) did not predict extubation success at 48 h in moderately to severely malnourished ESLD patients weaned from mechanical ventilation, decreased expiratory thickness of diaphragm assessed by ultrasound was associated with extubation failure.

Liberation from MV is a crucial part of caring for critically ill patients. Delaying extubation results in unnecessary discomfort, increased risk of complications, higher mortality, and increased costs of care.^2^ Despite the known pitfalls of prolonged invasive MV, up to 50% of the duration of MV is spent on the weaning process.^3^ Extubation and transition to negative pressure ventilation induces cardiac, pulmonary, neurologic and psychologic stress on a patient and even with the careful clinical assessment, >10% of extubations fail,^4^ and as many as 20% of extubated patients require reintubation within 72 h.^5^ Extubation failure is associated with prolonged MV, resulting in complications such as barotrauma, ventilator-associated pneumonia, ventilator-induced diaphragmatic atrophy, extended ICU stays and higher hospital mortality.^5,6^ Evaluation of a patient's readiness to be weaned from invasive MV includes assessment of numerous clinical and ventilator-derived parameters. Spontaneous breathing trial (SBT) is one of the major diagnostic tests to determine if patients can be successfully extubated and serve as a tolerance test, allowing those who do not experience neurologic, respiratory, or hemodynamic instability to be candidates for extubation.^7,8^ Rapid-shallow breathing index, respiratory rate, tidal volumes, negative inspiratory force (NIF), secretions amount and gag reflex, muscle strength assessment, or ability to follow commands represent some of the routinely used parameters allowing for a better assessment of the ability to tolerate extubation.^7^

We focused our attention on the special population of patients weaned from MV who are diagnosed with ESLD, a highly catabolic state significantly impacting muscle mass and leading to significant malnourishment and clinical deconditioning. This population of patients represents a challenge in critical care settings due to increased extubation failure rates and high mortality.^14^ Classic extubation parameters reflecting pulmonary mechanics at the time of extubation may not entirely reflect the patient's readiness to tolerate extubation for extended periods of time. In a study by Sasso et al,^14^ only 36% of extubated patients with cirrhosis avoided re-intubation at 72 h. Besides factors such as presence of infection or need for vasopressors, nutritional factors also directly influence the readiness for extubation. Malnutrition is very common in chronic liver disease and is seen in about 20% of patients with compensated cirrhosis and in up to 60% of those with advanced liver disease.^25^ A recent study focused on successful weaning from MV showed that high-calorie and protein-based nutritional supplementation was associated with a higher probability of successful ventilator weaning in patients undergoing prolonged MV.^26^ While nutritional surrogates such as prealbumin can identify patients with catabolic illness and are measured routinely, they are not always responsive to nutrient intake and therefore should not be used to detect malnourishment in critically ill patients.^27^

Ultrasound evaluation of diaphragmatic excursion and thickness at end inspiration has recently gained traction^28^ as a simple and useful tool to assess extubation readiness in patients who passed the SBT. During assisted MV, diaphragmatic thickening fraction (ΔTDI%) has been found to be an accurate index of respiratory muscle workload.^29^ Diaphragmatic thickening measurements have been used to predict extubation success or failure with optimal cutoffs ranging from 30% to 36%.^29,30^ A change in diaphragmatic thickening fraction (ΔTDI%) of at least 30% has demonstrated a sensitivity of 88% and specificity of 71% for extubation success.^11^ When incorporated with traditional clinical and physiologic measures to determine the timing of extubation, point-of-care ultrasound has been used to expedite extubation in patients with normal ΔTDI%.^31^ Time from ultrasound to extubation has been shown to be shorter in subjects with a normally functioning diaphragm (ΔTDI% ≥ 30%) compared to those with diaphragm dysfunction (ΔTDI% < 30%).^31^

In this study, we sought to explore and potentially expand the predictability of extubation success in the population of intubated and mechanically ventilated patients with end-stage liver disease who are moderately or severely malnourished by including POCUS in the process of weaning from MV. To our knowledge, this is the first prospective study evaluating ultrasonographic assessment of the diaphragm in the population of mechanically ventilated malnourished patients with ESLD, and we report several relevant findings that merit further discussion.

First, our study demonstrates that ultrasonographic assessment of diaphragm is feasible as diaphragm was clearly identified in all enrolled patients. In fact, the prevalence of ascites in patients with cirrhosis may allow for easier ultrasonographic visualization and measurement of diaphragm. Second, in contrast to several previous studies assessing diaphragmatic thickening fraction (ΔTDI%) in patients admitted to medical ICUs,^9–11,23,32–34^ our study did not show significant differences in ΔTDI% between patients who tolerated extubation (ET) and those who failed (EF). Nevertheless, the thickness of diaphragm during expiration, *T*_exp_, may be predictive of extubation success as it was significantly associated with the ability to remain extubated at 48 h. This finding should not come as a surprise given that thinning of the diaphragm reflects debilitation and can be measured accurately during maximal expiration, whereas the thickening fraction reflects the dynamic change of diaphragm and is directly related to the patient's effort and the ability to achieve maximal inspiration. Third, we found that conventional assessments for extubation readiness have limited ability to predict ET in this patient population, also identifying several readily observable clinical risk factors for extubation failure specific to patients with ESLD.

The presence of large ascites was associated with the risk of EF even after adjustment for relevant covariates. While the diaphragm thickness did not differ between those with severe versus those without severe ascites (data not reported here), since the presence of significant hydrothorax had a numeric trend toward EF, it is reasonable to conclude that fluid overload may add to the risks of EF in this cohort of patients. In particular, the increase in intrabdominal pressure from ascites is associated with decreased lung volumes from impaired diaphragmatic excursion, consequently causing rapid shallow breathing, and ultimately increased resting oxygen consumption; it may also lead to atelectasis of lung bases, increase in pleural effusion size and development of positive end-expiratory pressure with increased activation of inspiratory muscles, all of which may potentially compromise successful extubation.^35^

Along with the evaluation of POCUS-derived weaning parameters, our study evaluated the usefulness of other traditional clinical and ventilator-derived predictor of successful extubation. Two findings are notable—although RSBI and NIF statistically correlate with diaphragm thickening fraction, this correlation remains relatively weak. Additionally, among all evaluated parameters, commonly used RSBI or NIF were less informative compared to diaphragm expiratory thickness and even the subjective head-lift or leg-raise tests. In fact, the inability to perform a head-lift test was one of the few muscle strength tests associated with increased EF in a cohort of patients recovering from neuromuscular weakness following myasthenia crises.^36^ As opposed to NIF, maximal expiratory pressure (MEP), tidal volumes, RSBI, and ultrasonographic ΔTDI% all of which reflect specifically respiratory muscle strength, direct assessment of overall strength (eg ability to mobilize head and extremities quantified by the validated Medical Research Council (MRC) Muscle Strength Grading Scale) provides general insight into the degree of global debilitation which, especially in this cohort, may impact extubation failure^37,38^

This study has several limitations. A formal sample size calculation was not performed prior to the study thus this study was not powered to give a definite answer to possible associations between ultrasonographic diaphragm parameters and extubation success. Additionally, as we only included patients with cirrhosis and ESLD, our results may not be applicable to general critical care patients. Since ultrasound measurements of the diaphragm were taken 12 h prior to extubation, these measurements may not reflect the exact conditions immediately prior to extubation. Despite protocolized coaching, the inspiratory efforts may not have been equal among all patients. Enrolled patients were nonconsecutive which could imply a certain degree of selection bias. Protocolized lung ultrasound as an add-on to the diaphragm assessment was not performed, although it is possible that it could add to a more reliable interpretation of the diaphragm thickening findings interpretation. Although the eligibility criteria required the exclusion of patients with known diaphragmatic dysfunction at the enrollment, the majority of enrolled patients did not have previous formal diaphragm ultrasound to confirm normal diaphragm function. This study was not adequately powered to detect extubation failure as we enrolled a relatively small number of subjects which was further decreased as we excluded patients who were re-intubated for nonrespiratory reasons. Despite studying a high-risk population, the EF rate in this study did not differ significantly from previously reported EF rates in general critical care settings.^39^ To our knowledge, this study is the very first to evaluate ultrasonographic measures of the diaphragm to predict extubation failure in pre-OLT, malnourished ESLD patients. The images were obtained by an experienced operator via a standardized protocol with a collection of robust clinical and ventilatory weaning parameters. Confounding factors, including the presence of pleural/peritoneal fluid, degree of malnutrition, and total duration of ventilation, were all taken into consideration when assessing associations with clinical outcomes.

## Conclusions

Although diaphragm thickening fraction (ΔTDI%) did not predict extubation success at 48 h in moderately to severely malnourished ESLD patients weaned from mechanical ventilation, decreased expiratory thickness of diaphragm assessed by ultrasound was associated with extubation failure. Larger and randomized trials are needed to better elucidate appropriate use of diaphragm ultrasound during extubation of high-risk, malnourished patients.

## Supplemental Material

sj-doc-1-cra-10.1177_29768675251337833 - Supplemental material for Point-of-Care Ultrasound Evaluation of Diaphragm as a Predictor of Extubation Success in Mechanically Ventilated, Malnourished, End-Stage Liver Disease Pretransplant Candidates—Observational Cohort Study Supplemental material, sj-doc-1-cra-10.1177_29768675251337833 for Point-of-Care Ultrasound Evaluation of Diaphragm as a Predictor of Extubation Success in Mechanically Ventilated, Malnourished, End-Stage Liver Disease Pretransplant Candidates—Observational Cohort Study by Adam Muhammad Reid, Sarathi Bhattacharyya, Zian Zhuang, Nida Qadir, Amy Schnabel, Alan Chiem, Semi Yoon, George Lim and Igor Barjaktarevic in Therapeutic Advances in Pulmonary and Critical Care Medicine
